# Revesz syndrome revisited

**DOI:** 10.1186/s13023-020-01553-y

**Published:** 2020-10-23

**Authors:** Michael Karremann, Eva Neumaier-Probst, Frank Schlichtenbrede, Fabian Beier, Tim H. Brümmendorf, Friedrich W. Cremer, Peter Bader, Matthias Dürken

**Affiliations:** 1 Department of Pediatrics, University Medical Center Mannheim, Theodor-Kutzer-Ufer 1-3, 68167 Mannheim, Germany; 2 Department of Neuroradiology, University Medical Center Mannheim, Mannheim, Germany; 3 Department of Ophthalmology, University Medical Center Mannheim, Mannheim, Germany; 4 Department of Hematology and Oncology, University Hospital of RWTH Aachen, Aachen, Germany; 5 SYNLAB Center for Human Genetics Mannheim, Mannheim, Germany; 6 Department of Pediatrics, Pediatric Stem Cell Transplantation, University Hospital Frankfurt, Frankfurt, Germany

**Keywords:** Bone marrow failure, Cerebellar hypoplasia, Exudative retinopathy, Growth retardation, Pancytopenia, Revesz syndrome, Shelterin, Telomere, TINF2

## Abstract

**Background:**

Revesz syndrome (RS) is an extremely rare variant of dyskeratosis congenita (DKC) with only anecdotal reports in the literature.

**Methods:**

To further characterize the typical features and natural course of the disease, we screened the English literature and summarized the clinical and epidemiological features of previously published RS cases. In addition, we herein describe the first recorded patient in central Europe.

**Results:**

The literature review included 18 children. Clinical features are summarized, indicating a low prevalence of the classical DKC triad. All patients experienced early bone marrow failure, in most cases within the second year of life (median age 1.5 years; 95% CI 1.4–1.6). Retinopathy occurred typically between 6 and 18 months of age (median age 1.1 years; 95% CI 0.7–1.5). The incidence of seizures was low and was present in an estimated 20% of patients. The onset of seizures was exclusively during early childhood. The Kaplan–Meier estimate of survival was dismal (median survival 6.5 years; 95% CI 3.6–9.4), and none of the patients survived beyond the age of 12 years. Stem cell transplantation (SCT) was performed in eight children, and after a median of 22 months from SCT four of these patients were alive at the last follow up visit.

**Conclusion:**

RS is a severe variant of DKC with early bone marrow failure and retinopathy in all patients. Survival is dismal, but stem cell transplantation may be performed successfully and might improve prognosis in the future.

## Background

Dyskeratosis congenita (DKC) is a multisystem disorder classically defined by a triad of clinical symptoms including oral leukoplakia, hyperpigmented reticular skin lesions, and nail dystrophy, but the clinical features are highly variable [1, 2]. Individuals are prone to develop bone marrow failure, malignancies, immunodeficiency, and pulmonary complications. Less common features include dental and eye abnormalities, esophageal stenosis, urethral stenosis, avascular necrosis of the femur and/or humerus, osteopenia, enteropathy, and liver disease [3]. Within this wide spectrum of clinical phenotypes, some rare entities represent distinct variants of DKC, namely Høyeraal-Hreidarsson syndrome (HHS, OMIM #305000) and Revesz syndrome (RS; OMIM #268130) [4]. Cerebroretinal microangiopathy with calcifications and cysts (CRMCC; formerly Coats plus syndrome, OMIM #612199) is another disorder of telomere maintenance, with distinct overlap to RS in clinical presentation [5].

Høyeraal-Hreidarsson syndrome is a severe variant of DKC [6]. Patients typically harbor intrauterine growth retardation, microcephaly, and cerebellar hypoplasia. The further course of disease is characterized by developmental delay, and early, progressive bone marrow failure, with the latter leading to death during childhood [7, 8]. First described in 1988 by Tolmie et al. [9, 10], extensive intracranial calcifications, leukodystrophy, and bilateral exudative retinopathy characterize CRMCC. Other features, including bone marrow failure during adolescence and early adulthood, increased risk of intestinal bleeding and bony lesions have been described [11–14], and may help to clinically discriminate CRMCC from RS [15]. The latter entity was first described in 1992 [16], and is characterized by early bone marrow failure and bilateral exudative retinopathy. It features skin, hair, and nail abnormalities, intracerebral calcifications, cerebellar hypoplasia, and ataxia. In addition, children may be born small for gestational age and exhibit developmental delay. Death usually occurs during early childhood [17–20].

All variants of DKC share dysfunctional telomere maintenance, resulting in a reduced protection from cellular senescence and accelerated exhaustion of (in particular) high turnover tissues, genetic instability, and increased frequency of secondary malignancy. To date, a minimum of 11 genes of the DKC complex have been shown to harbor mutations accounting for the different subtypes of DKC. However, the association of genotype and phenotype is highly variable. While patients with classical DKC harbor mutations in *ACD*, *CTC1*, *DKC1*, *NHP2*, *NOP10*, *PARN*, *RTEL1*, *TERC*, *TERT*, *TINF2*, and *WRAP53* [4], several of these mutations may also underlay the more severe HHS [6, 21]. In contrast, CRMCC and RS are linked to mutations in distinct genes, namely the CST telomere replication complex component 1 (*CTC1*), and in the TRF1-interacting nuclear factor-2 (*TINF2*), respectively [22, 23].

In RS, mutations in *TINF2* at chromosome 14q11.2 result in dysfunctional TIN2 protein leading to impaired telomere protection by the shelterin complex [24]. Patients harbor extremely short telomeres, even compared to patients with classical DKC [3, 20]. Mutations in additional genes reported in DKC patients affecting the telomerase complex have not been associated with RS. Nevertheless, the same mutations in *TINF2* may be found in classical DKC patients presenting clinically without the distinct RS phenotype, hence, the genotype alone is not able to define RS [20, 25, 26]. Discriminating RS from other variants solely by clinical criteria may also be challenging. Furthermore, the clinical features vary significantly in individual patients, and overlap of RS with other DKC variants [27, 28] or alternate disease entities, such as Fanconi anemia or others, is common [29–32]. In addition, accelerated telomere shortening can also be observed in non-hereditary clonal and non-clonal disorders affecting individual (mostly the hematopoietic) stem cell compartments [33].

To the best of our knowledge, no more than 17 patients with RS, together with sufficient clinical data, have been published in the English literature to date [3, 16–20, 23, 34–42]. Reviewing these cases and reporting the first patient in central Europe, the present work aimed to further characterize this rare entity.

## Patients and methods

### Mutational and telomere length analysis

The average length of telomere repeats in peripheral blood lymphocytes and granulocytes of the patient (at the age of 15 months), as well as her parents and siblings, was determined by flow-FISH as previously described [43]. Molecular analysis included screening for genetic alterations within exon six of the *TINF2* gene.

### Literature search

To identify previously published patients with RS, an electronic *PubMed* search of the English literature was performed using the terms “bone marrow failure”, “aplastic anemia”, and “pancytopenia”, each in combination with “retinopathy”. In addition, *PubMed* was searched for “Revesz syndrome”, and “TINF2”. Further case reports were included, if referred to in the relevant literature. In case of insufficient clinical information within the publications, the respective corresponding authors were contacted and requested to provide missing data. Individuals who provided further information are mentioned in the acknowledgement section.

### Statistical analyses

Statistical analysis was performed using IBM SPSS Statistics® version 25 (IBM, Armonk, NY, USA). Survival, as well as the time-dependent cumulative risk to develop bone marrow failure, retinopathy, and seizures, was estimated by Kaplan–Meier analysis. Patients were censored in case of death, and at the age of the last reported visit. All patients were defined regarding gender (male/female), and potential features of RS (present/absent/unknown). A chi^2^-test was applied to compare the sex of patients to an equal gender distribution. Due to the small number of patients, the retrospective character of the study, and a potential bias since the underlying case reports might not be representative, the prevalence of these features was discussed cautiously. Statistical numbers are intended to be “hypotheses generating”. Details are given in Table 1.

**Table 1:** Summary of clinical findings and outcome in 18 patients with RS

	Ratio ± or m/f (number of patients with information)	Duprey and Steger [35]	Revesz et al. [16]	Kajtar and Mehes [36]	Riyaz et al. [39]	Scheinfeld et al. [23]	Negron et al. [38]	Savage and Bertuch [3]	Sasa et al. [20]	Asai et al. [34]	McElnea et al. [37]	Tamura et al. [40]#	Gupta et al. [17]	Gupta et al. [17]	Moussa et al. [18]	Tomcikova et al. [41] #	Watanabe et al. [42]	Sakwit et al. [19]	This report
*Patient number*		1	2	3	4	5	6	7	8	9	10	11	12	13	14	15	16	17	18
*Gender (m/f)*	11/7 (18)	f	m	f	f	m	m	m	m	m	m	m	f	f	m	m	f	m	f
*Medical history*																			
Preterm	4/12 (16)	–	–	–	–	–	–	?	–	–	+	–	+	+	?	–	?	?	+
Small for gestational age	9/6 (15)	+	+	+	+	–	+	?	–	+	–	+	–	–	?	+	+	?	–
Failure to thrive	6/4 (10)	+	–	–	+	–	–	?	+	?	?	?	?	?	?	?	+	+	+
*Neurologic findings*																			
Microcephaly	6/5 (11)	+	–	+	+	–	–	?	+	?	?	–	?	?	?	–	+	?	+
Cerebellar hypoplasia	13/4 (17)	–	+	+	?	+	–	+	+	–	+	+	+	+	+	–	+	+	+
Cerebral calcifications	11/2 (13)	+	+	–	?	+	+	?	?	+	?	–	+	+	+	+	?	+	+
Ataxia	4/5 (9)	–	+	–	?	–	–	?	+	?	?	+	?	?	?	–	?	?	+
Seizures	3/14 (17)	+	–	–	–	–	–	?	–	–	–	–	–	–	–	–	–	+	+
Neurodevelopmental delay	12/5 (17)	+	+	–	+	+	–	+	+	–	+	+	–	–	+	+	?	+	+
*Ophthalmologic findings*																			
Exudative retinopathy	18/0 (18)	+	+	+	+	+	+	+	+	+	+	+	+	+	+	+	+	+	+
Glaucoma	3/11 (14)	–	–	+	–	–	+	?	–	–	–	?	–	–	–	+	?	–	?
Bone marrow failure	18/0 (18)	+	+	+	+	+	+	+	+	+	+	+	+	+	+	+	+	+	+
*Dermatologic findings*																			
Irregular nails	13/2 (15)	+	–	+	+	+	–	+	+	+	+	+	?	?	+	+	?	+	+
Skin findings	6/5 (11)	+	+	–	+	–	–	+	?	–	?	+	?	?	?	+	?	?	–
Leukoplakia	6/8 (14)	–	–	+	+	–	–	+	+	–	–	–	?	?	?	–	?	+	+
Fine, sparse hair	4/4 (8)	–	+	+	+	–	?	?	?	?	?	–	?	?	?	–	?	?	+
*Therapy and outcome*																			
Stem cell transplantation	8/12 (18)	No	No	Yes	No	No	No	Yes	Yes	Yes	No	Yes	No	No	Yes	No	Yes	No	Yes
Last follow up (age/years)	–	4	1.6	3.5	6	5	12	6.8	2.0	3.5	1.2	6.5	1.5	1.5	3.3	7	3.4	2.7	4.2
Alive	10/6 (18)	Yes	No	Yes	No	Yes	No	No	No	Yes	Yes	No	Yes	Yes	Yes	Yes	Yes	No	No
*Genetic TINF2 alteration*	12/0 (12)	?	?	?	?	?	?	p.R282H	p.K280RfsX36	p.R282H	p.R282H	p.R282H	p.T284P	p.T284P	p.R282H	p.P289S	p.R282H	p.T284Nfsx6	p.R282H

+ prevalent, – absent, ? not determined, # additional information was provided by personal communication

## Results

### Case report

The female infant was delivered preterm after 33 gestational weeks (birth weight 1360 g, P10) as the 4th child of non-consanguineous parents with Caucasian descent. Apart from a mild oligohydramnios and suspected growth retardation, pregnancy was uneventful. Delivery and postnatal course were without complication and, in particular, no respiratory support was required.

**Fig. 1:**
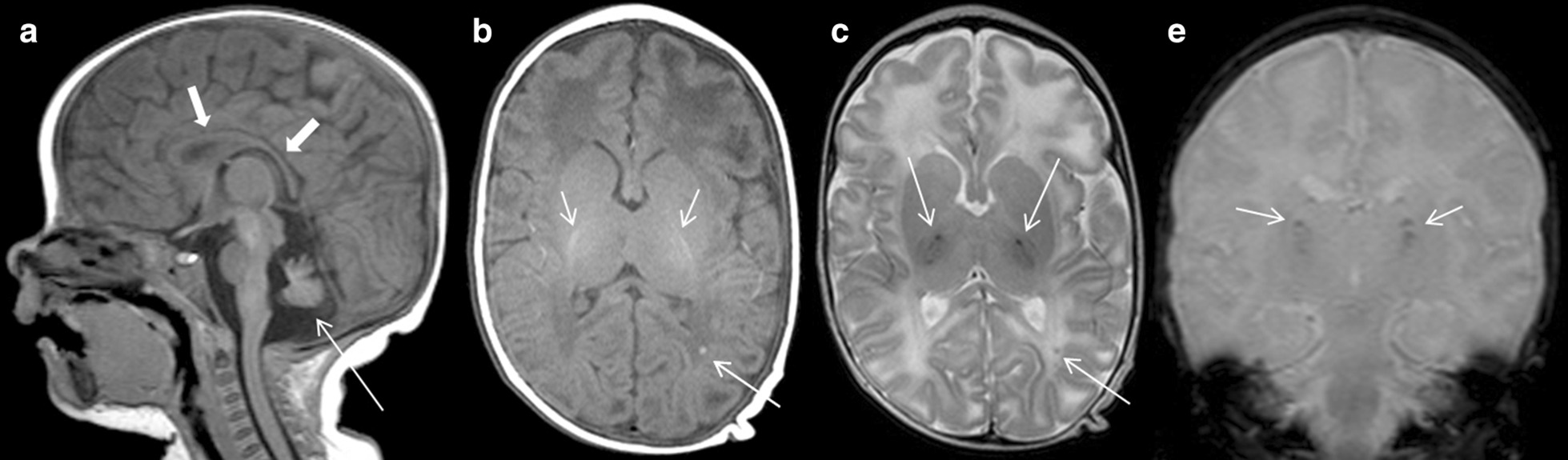
Initial MRI at the age of 2.5 months performed after the first partial seizure. Sagittal T1-weighted MRI image demonstrates cerebellar hypoplasia with an emphasis on the vermis and tonsils (arrow) and consecutive enlargement of the cerebrospinal fluid (CSF) spaces. The corpus callosum has a normal shape and size (thick arrow) (**a**). Transversal T1-weighted MRI image shows normal myelination within the internal capsule concealing the punctuate high signal (**b**) corresponding to the low signal areas in the T2-weighted (**c**) and T2*-weighted images within the thalami (**d**). These areas of signal abnormality correspond to calcifications. Also note the abnormal signal intensity in the frontal lobes (**b**, **c**) corresponding to leukencephalopathic areas also seen in the follow ups. At this point there was no administration of gadolinium contrast material

At the age of 2½ months, the girl experienced her first partial seizure following vaccination. MRI imaging revealed cerebellar hypoplasia and periventricular white matter changes, at this time attributed to prenatal hypoxemia (Fig. 1). Due to recurrent seizures, an anticonvulsive treatment with levetiracetam and topiramate was initiated. Pancytopenia was first seen at the age of 9 months, during an episode of bronchitis (leukocytes 2600/µL, hemoglobin 8.7 g/dL, thrombocytes 15,000/µL). Over the next few months, the blood count continued to worsen to very severe neutropenia (absolute neutrophil count < 200/µL), resulting in platelet transfusions twice weekly and erythrocyte transfusions every other week. Diagnostic workup showed bone marrow aplasia without signs of myelodysplasia or cytogenetic abnormalities. Metabolic diseases and infections, as well as Fanconi anemia and paroxysmal nocturnal hemoglobinuria, were ruled out as the underlying pathology for the observed bone marrow failure.

**Fig. 2:**
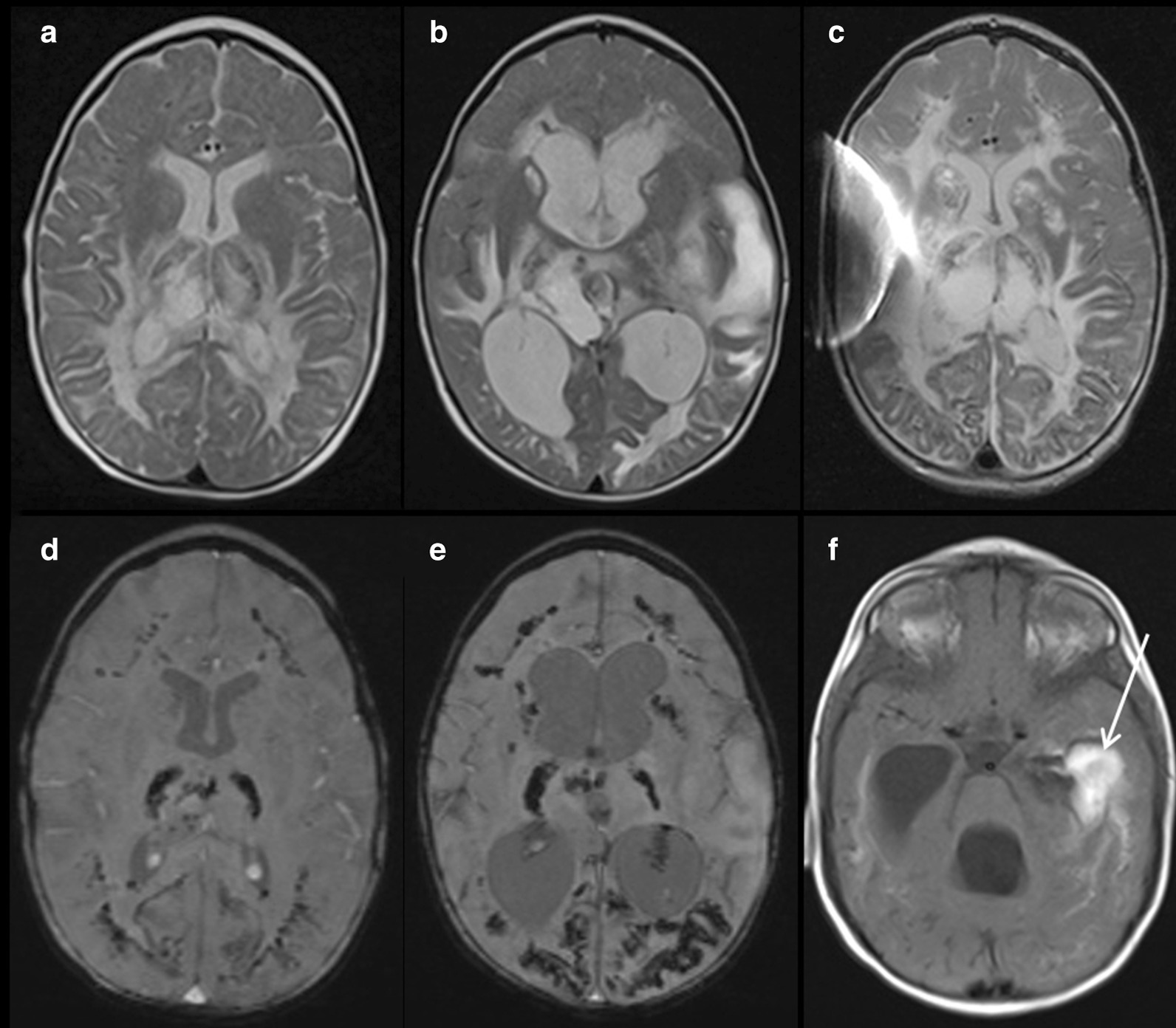
Axial T2-weighted images demonstrate progression of symmetric white matter lesions periventricular, within the thalamus, in the internal capsule, and the basal ganglia at the age of 1 year (**a**), 2 years (**b**), and 3.8 years (**c**). The corresponding axial susceptibility-weighted images (SWI) demonstrated progressive foci with susceptibility artefacts distributed symmetrically within the basal ganglia, the thalami, and on the border between gray and white matter (**d**, **e**), corresponding to calcifications. At the age of 2 years the patient suffered from a hemorrhage at the level of the left temporal horn with intraventricular breakthrough, shown as an area of high intensity (arrow) on the T1-weighted image (**f**), and hydrocephalic congestion (**b**, **f**), resulting in the need for ventricular-peritoneal shunt insertion. The artefact on the right side is due to the implanted shunt (**c**)

Soon after birth, an exudative retinopathy had been diagnosed and treated with repeated intraocular Bevacizumab injections. Furthermore, the patient exhibited a mild psychomotor developmental delay and a failure to thrive, e. g. body weight of 5 kg (< P3), length of 62 cm (< P3), and head circumference of 40 cm (< P3) at the age of 10 months. In contrast to her siblings, she had fine, sparse hair, but normal skin and nails (nail dystrophy developed by the age of 4 years). A second MRI of the brain at the age of 1 year confirmed cerebellar hypoplasia, but also revealed multiple demyelinating areas, both in the periventricular and subcortical white matter, in part with calcifications (Fig. 2 and Additional file 1: Figure 1). In addition, a distinct hypoplasia of the corpus callosum developed, most likely due to increasing leukoencephalopathy (Additional file 2: Figure 2).

**Fig. 3:**
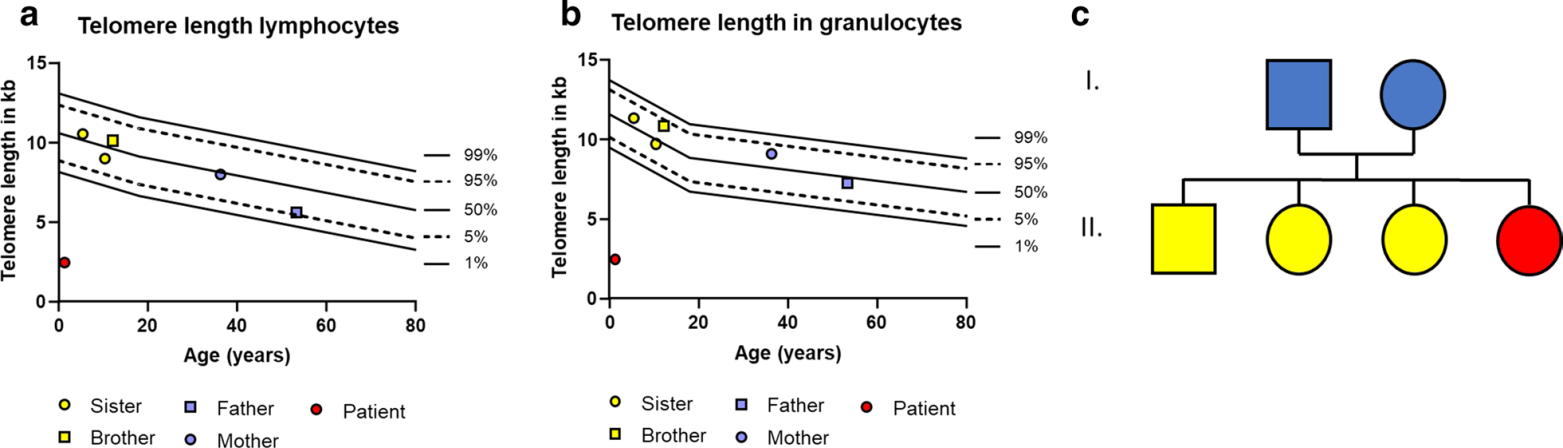
Telomere length analysis of the patient (red), her siblings (yellow), and parents (blue) demonstrates the shortening in the patient’s **a** lymphocytes and **b** granulocytes, substantially below the first percentile of age. In contrast, the results of her parents and siblings were within normal limits. **c** Pedigree. Circle indicates female, square indicates male gender

The diagnosis of RS was finally suspected based on of the clinical signs and MRI findings. This was confirmed by telomere length analysis, which revealed extremely short telomeres of 2.47 kilobases (kb) in peripheral blood lymphocytes and 2.48 kb in peripheral blood granulocytes, both substantially below the first percentile of healthy controls. Both parents and three siblings exhibited telomere lengths within the normal range (Fig. 3). Mutation analysis detected a genetic alteration in exon six of *TINF2* with a previously published base change c.845G > A, leading to the amino acid change Arg282His.

The child underwent allogeneic bone marrow transplantation (SCT) from a healthy HLA-identical sibling at the age of 17 months. The conditioning regimen included fludarabine (30 mg/m^2^ day − 7 to − 3), cyclophosphamide (30 mg/kg day − 6 to − 3), and anti-thymocyte globulin (20 mg/kg day − 4 to − 2). Apart from an invasive aspergillosis of the lungs and a mild acute graft-versus-host disease, no serious complications occurred. Hematological engraftment was achieved after day + 11, and day + 55 for granulocytes and thrombocytes, respectively. The girl exhibited an early T-cell expansion with > 500 CD3 + cells/mL at day + 60. A reactivation of Adenovirus detected by PCR at day + 70 resolved spontaneously. Aspergillus infection was treated with intravenous liposomal amphotericin B and caspofungin, followed by an oral treatment with voriconcazole until 10 months from SCT, resulting in residual lesions in both upper pulmonary lobes. Further mild and non-persisting complications within the first year from SCT included Epstein-Barr virus reactivation (treated by one single infusion of rituximab), and persisting mild thrombocytopenia, most likely attributed to anticonvulsive treatment.

During the second year after SCT, the girl experienced progressive abdominal pain crises resulting predominantly in malnutrition and further failure to thrive. The cause of these could not be clarified, but graft versus host disease of the gut was ruled out. In addition, a spontaneous intraventricular hemorrhage occurred at the age of 2 years, finally resulting in hydrocephalus and a need for ventricular-peritoneal shunting (Fig. 2f). At this time, blood count and hemostaseologic parameters were within normal limits. Therefore, we propose that this bleeding event was most likely due to an increased intracerebral pressure during extensive crying, and perhaps fostered by an increased fragility of the intracerebral vessels.

During the girl’s 3rd and 4th year of life, psychomotor delay remained despite extensive training. Visual acuity worsened, resulting in a nearly complete loss of vision at the age of 3 years (corresponding retinal detachment shown in Additional file 3: Figure 3). Also, somatic development was seriously delayed. At the age of 4 years, the patient experienced severe aspiration pneumonia, and finally passed away due to refractory lung failure.

### Literature search

In addition to the patient reported in this work, a literature search revealed 22 cases of RS. Five patients were excluded, since only very limited clinical data were available. Additional information concerning missing clinical data within the references was provided by the respective authors of the case reports on request for patient #11 and #15. Patient #1, with features of RS published in 1988, was included, even though this was published prior to the characterization and definition of Revesz. Thus, 17 patients from the literature were eligible for evaluation.

### Patients characteristics

In total, this study reviewed 18 cases of RS (Table 1); seven females and 11 males, resulting in a male predominance of 1.6:1 (not significant). Of note, stigmata belonging to the classical clinical DKC triad of oral leukoplakia, hyperpigmented skin findings, and nail dystrophy were prevalent in only 43% (n = 6/14), 55% (n = 6/11), and 87% (n = 13/15) of patients with information, respectively. In contrast to classical DKC, all cases of RS represented de novo mutations with unaffected parents, reflecting the severe course and early death in this disease.

**Fig. 4:**
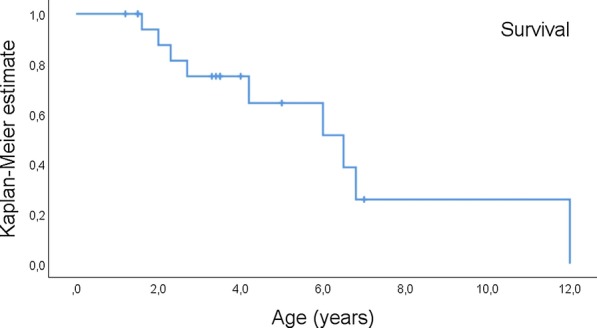
Kaplan–Meier estimate of survival in 18 patients with Revesz syndrome. Median survival was 6.5 years (95% CI 3.6–9.4)

**Fig. 5:**
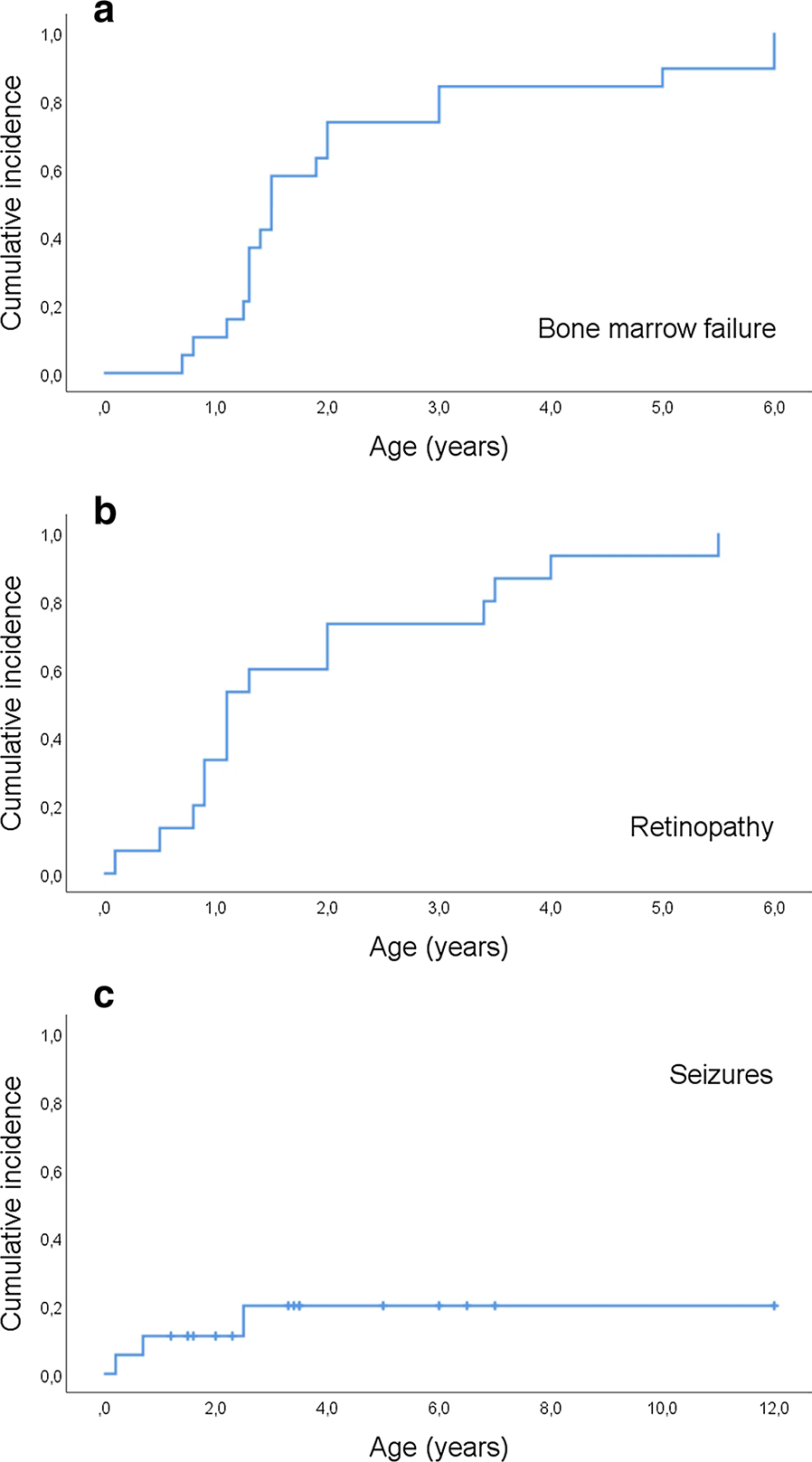
Estimated cumulative incidence of various features of RS: **a** All patients developed bone marrow failure (BMF) by the age of 6 years. Time of diagnosis was available in 16 patients, with BMF occurring most frequently in the second year of life (median age 1.5 years; 95% CI 1.4–1.6). **b** Accordingly, retinopathy was present in all cases (time of diagnosis was available in 15 patients). Most children developed retinopathy between the ages of 6 and 18 months of life (median age 1.1 years; 95% CI 0.7–1.5). **c** Only three patients (unknown in two) experienced seizures. These developed exclusively during early childhood, resulting in an estimated cumulative incidence rate of 20% (SD 10.7)

The very severe prognosis of RS could be substantiated in the present work. Kaplan–Meier analysis estimated a median survival of only 6.5 years (95% CI 3.6–9.4), and no patient beyond the age of 12 years was identified in the literature (Fig. 4). Patients died from BMF in most cases, but refractory lung failure was another cause of death in some cases. Bone marrow failure occurred in all patients between birth and the age of 6 years, in most cases within the second year of life (median age 1.5 years; 95% CI 1.4–1.6, Fig. 5a). BMF lead to stem cell transplantation in eight patients, four of whom were still alive at the end of follow up 1 month (#3), 20 months (#9), and 2 years (#14 and #16) from SCT. Details on the conditioning regimen were specified in four patients; all underwent a reduced conditioning regimen, including fludarabine (100–180 mg/m^2^), cyclophosphamide (100–120 mg/kg), and anti-thymocyte globulin. Patients with this regimen tolerated SCT well without severe early transplantation-related toxicity. All patients developed retinopathy, most likely between the ages of 6 and 18 months (median age 1.1 years; 95% CI 0.7–1.5; Fig. 5b). Despite treatment, retinopathy led to a severe loss of visual acuity in most patients. Glaucoma occurred in three cases. Frequent features of RS include neurological and neuroanatomical abnormalities. In the present cohort, cerebellar hypoplasia (76%, n = 13/17) and intracranial calcifications (85%, n = 11/13) were common features, whereas microcephalia and ataxia were reported less often (Table 1). Only three patients (unknown in one) experienced seizures, that developed exclusively during early childhood, resulting in a cumulative incidence rate of 20% (SD 10.7; Fig. 5c). Our study substantiated neurodevelopmental delay and/or mental retardation to be a frequent feature of RS, and this was present in 71% of patients (n = 12/17), frequently emerging during infancy. However, learning deficits were often mild, and absent in others, indicating that neurocognitive impairment is not an obligatory feature of the syndrome. Of note, intracranial hemorrhage was detected in three patients during the course of the disease and might represent a yet underrecognized cerebrovascular vulnerability. Patients with RS are born small for gestational age and/or preterm, resulting in a mean birth weight of 1950 g ± 410 (SD), and one out of three patients failed to thrive (Table 1).

Genetic mutational analysis was available in 66% of patients (n = 12) and was performed in all patients that were published since 2010. All of these harbored a *TINF2* mutation and the underlying alterations were restricted to exon six. The most frequent mutation was c.845G > A, a missense mutation resulting in an amino acid substitution of Alanine to Histidine at position 282 of the TIN2 protein. Further mutations included two heterozygous deletions resulting in a frameshift and premature stop codon leading to truncated TIN2 proteins (c.839delA, p.K280RfsX36, patient #9; and c.851-855 del CAGTC; p.T284Nfsx, patient #17) and alternate missense mutations at position 284 (c.850A > C; p.T284P; patients #12, 13) and 289 (c.865C > T, p.P289S, patient #15) of the TIN2 protein. Hence, all mutations were located between amino acid 280 and 289.

Telomere analysis was available in seven patients. All presented with very short lengths, reported as “below the first percentile for their age” (patients #10, #12, #13, #20), reported in absolute numbers of 2.3–2.8 kb (#8) and 2.5 kb (#20) and/or given as relative shortening of − 5.7 SD (#16).

## Discussion

Revesz syndrome is an extremely rare disorder, and to date, patients have only been published anecdotally. Therefore, the present work aimed at further characterization of this rare entity by summarizing the clinical findings of 17 previously published cases of RS [3, 16–20, 23, 34–42], and presenting the one further case reported in this work. The latter represents the first published patient from central Europe.

### Medical history and clinical signs

In the present cohort, we found a slight and non-significant male predominance. This is far less pronounced compared to DKC, with its often X-linked inheritance [2]. Given the autosomal background in RS, we would agree with Gupta and coworkers who state an equal gender distribution in RS [17]. In all, our findings argue against previous presumptions of a considerable male predominance of 3:1 [18].

Looking into the patients’ medical history, growth retardation, either prevalent at birth or a failure to thrive was prevalent in most cases. Four children were delivered preterm, and of note, those did not present small for gestational age. Unfortunately, information on further somatic development was available for only one of these children, and this girl experienced severe failure to thrive soon after birth, indicating that growth retardation starts during late pregnancy or postnatally. However, some children regained underweight. Hence, growth retardation is common, but not a general trait in patients with RS.

Nail dystrophy was the most frequent feature of the clinical DKC triad. In line with findings in classical DKC, typical skin pigmentation and oral leukoplakia were even less frequent [44], resulting in the complete clinical triad in only two cases, in contrast to 35% in DKC [44]; hence, the clinical signs of the DKC triad seem less common in patients with RS [45]. This might be confusing, since the prevalence of these correlate with a more severe course of the disease and early onset BMF in classical DKC [4, 44]. However, the seemingly low incidence in RS is in line with previous assumptions [26] and might well be due to the young age at diagnosis and early death of these patients, since the DKC triad may develop with age and sometimes following BMF [4, 45] between the ages of five and 13 years [2, 46]. Accordingly, in patients #11 and #18 of our cohort, nail dystrophy was absent when the diagnosis of RS was made but developed during course of disease. Furthermore, this review might underestimate the prevalence, since findings may be subtle and not all case reports commented on these signs. We would therefore suggest careful screening for signs of the DKC triad in patients with suspected RS, not only at time of initial diagnosis but also at older ages. However, diagnosis of RS is based on additional features and a lack of DKC signs, especially in young children, should not preclude from providing a diagnosis [4].

As in DKC, patients with RS may be prone to developing secondary malignancies [47]. Most likely due to the short survival time, none of the patients from the present report experienced such complications. However, once survival might improve, patients should be monitored accordingly.

### Bone marrow failure

TINF2 mutations have been linked to severe BMF [48]. Accordingly, BMF, occurring at an even younger age than previously suspected, is a general trait of RS [18]. Reflecting the most severe course of disease, BMF was most likely to develop within the second year of life and, in all cases, developed by the age of 6 years (Fig. 5a).

Stem cell transplantation (SCT) was performed in eight patients from the present cohort, and four of these were still alive at the last documented visit. Hence, SCT should be offered to patients, if required clinically, potentially resulting in improved survival in the future. Various conditioning regimens, stem cell sources, and donors have been published [3, 18, 20, 34, 36, 40, 42]. The authors point out a relevant treatment-related toxicity due to the underlying telomere disorder (particularly affecting the lung), and the approach should take into account experiences in DKC patients. In this regard, Ostronoff suggested a reduced intensity conditioning (RIC) regimen including fludarabine, cyclophosphamide, and anti-thymocyte globulin in DKC patients [49]. Within the present cohort, four of the eight cases, including our patient, underwent this regimen with only limited acute SCT-associated toxicity [34, 40, 42]. Hence, we suggest applying this conditioning regimen in patients with RS, but still SCT in RS remains a high-risk transplantation and should, therefore, be carried out by experienced centers only.

### Pulmonary disease

Lung complications contribute to treatment-related mortality, especially following SCT in adult DKC patients [46, 48, 50]. Accordingly, two deaths in this series occurred due to terminal lung failure 2 years [this report] and 4.5 years [40] after the date of the successful SCT. Conditioning regimens with potential pulmonary toxicity should, therefore, be avoided to prevent worsening of occult lung disease [42, 51]. Given that pulmonary interstitial fibrosis is independently attributed to *TINF2*-mutations [49, 52, 53], lung disease due to insufficient telomere maintenance may be regarded as an obligatory late sequela in patients with RS also without SCT [52]. Accordingly, in DKC, sporadic lung disease may develop within the second and third decade, but with a median of 4.7 years following SCT [53]. Only one of the 10 cases without SCT in the present review experienced lung failure (at the age of 12 years), most likely due to the short survival time of the others [38]. Therefore, this complication should not preclude one from performing SCT, but patients should be educated and monitored accordingly. Potentially, early and aggressive interventions in case of respiratory symptoms might further improve survival (especially following SCT) in the future.

### Retinopathy

In the present study, exudative retinopathy occurred during infancy and early childhood, most likely between the ages of 6 and 18 months (Fig. 5b). Some children were born preterm, but the medical history was unlikely to determine an association between retinopathy and prematurity. Hence, RS should be considered in case of Coats-like retinopathy in infants without a typical history. Therapy included photocoagulation [17, 18, 20, 23, 34, 37, 41], repeated bevacizumab injections [17, 34], retinocryopexia [35, 36], and surgical approaches, including vitrectomy [17, 34, 37, 41] and enucleation, in cases of complete loss of visual acuity with or without painful glaucoma [16, 36, 41]. To date, laser photocoagulation is the preferred mode of therapy, and bevacizumab injections are an alternative [50]. However, in RS-associated retinopathy, most patients will develop a severe loss of visual acuity despite treatment.

Exudative retinopathy is a defining trait of RS [4]. However, there is a huge overlap to alternate entities that may harbor other features of RS, including BMF, failure to thrive, mental retardation, and the cerebral abnormalities. These include patients with DKC and potentially mutations other than *TINF2*, in whom retinopathy may occur occasionally [28, 51, 54, 55]. These patients may be discriminated from RS by the less severe course of disease and/or the absence of a *TINF2* mutation. In addition, in cases of a familial history of DKC, the diagnosis of RS should be questioned. HHS may be associated with retinopathy [27], mimicking the severe course of RS. However, a familial history of DKC and mutations outside TINF2 may exclude RS and classify these patients as HHS. Moreover, a relevant overlap is found with CRMCC. This disease, formerly called Coats plus syndrome, resembles RS regarding bilateral exudative retinopathy, intracranial calcifications, mucocutaneous abnormalities, and bone marrow failure. It can be discriminated by molecular analysis, since CRMCC is restricted to *CTC1* mutations. Clinically, it differs from RS by the prevalence of bony lesions and intestinal bleeding [11–14]. Since SCT is dispensable due to the late onset and often mild course of BMF, discriminating CRMCC from RS is of upmost importance [15]. Further disorders include Fanconi anemia [31, 32, 56] and rare entities, including mutations in *DNAJC21* and *ERCC6L2* [29, 30].

Conversely, patients with features of RS including early onset of BMF and *TINF2*-mutations but lacking exudative retinopathy are published in the literature. These cases differ from RS not only in terms of the ophthalmologic findings, but they also usually exhibit a less progressive course and longer survival. These patients should not be classified as RS [3], but represent variants of autosomal dominant DKC [57–59]. Therefore, accurate clinical evaluation, including verification or exclusion of retinopathy, is essential to classify the disease properly and thereby enabling clear decisions on best possible treatment of the patient.

### Central nervous system (CNS) manifestation

Cerebral calcifications is a central trait of RS [4, 46], reported in all but two patients with clinical information. Hence, even if calcifications may be seen in other entities, including CRMCC, HS, and DKC, this feature in combination with retinopathy, early onset of BMF, and the molecular evaluation of *TINF2* is a valuable predictor of RS. Therefore, diagnosis of RS in the absence of cerebral calcifications should be based on other strong criteria. Microcephaly and cerebellar hypoplasia were less common. The latter is usually attributed to HHS [21]. However, cerebral abnormalities are common in TBD and seem to widely overlap between the various DKC variants, with none being specific for a distinct entity [60]. In this regard, we describe the first patient with RS and a hypoplastic corpus callosum. Of note, given that the first MRI soon after birth revealed normal findings, we would hypothesize that the hypoplasia may be attributed to leukoencephalopathy, and the rarity of this trait in RS may be due to the short life expectancy in most patients. In this regard, involution of the corpus callosum may be a general feature in TBI, as it has been described in various patients with HHS [61]. Rarely, gliotic lesions may occur [23].

Of note, three patients of the present cohort experienced intracranial hemorrhages resulting in relevant morbidity [19, 38]. These bleeds might have been associated with cerebrovascular fragility rather than with low platelets or a plasmatic bleeding predisposition, since cerebral bleeding has been reported in other patients with *TINF2* mutations [58], and bleeding from telangiectasias including gastrointestinal hemorrhages is a common trait of other telomere biology disorders [50]. Negron and coworkers hypothesized that reabsorption and healing of such cerebral (micro)bleedings might result in calcifications and, hence, be a pathophysiological trigger of this frequent feature in RS [38].

Scheinfeld has summarized neuroradiologic findings in RS [23]. Herein, we provide further paradigmatic NMR imaging findings.

Neurodevelopmental delay is common in RS, but symptoms may be mild, with “satisfying” neurocognitive development [36], or absent in some patients. In fact, one of the patients was a “good student with above-average intelligence and a skillful violin player” [38]. However, early and repeated neurodevelopmental assessment is indispensable in patients with RS and adequate treatment is essential in cases of pathological delay. Despite the numerous cerebral abnormalities, only few patients experienced seizures, and these developed exclusively during early childhood (Fig. 5c). Hence, in line with other telomere biology disorders [46], epilepsy may occur occasionally, but is not a typical feature of RS.

### Genetic background and telomere biology

RS is a telomere biology disorder (TBD) within the DKC spectrum resulting in impaired telomere maintenance [62]. The principles of TBD have been summarized by Barbaro, who focused on the various clinical entities [46], whereas Smith has extensively reviewed the biochemical background of telomere maintenance [24].

In contrast to DKC, RS is restricted to mutations in one single gene, namely *TINF2* on chromosome 14q12 [46]. Five different mutations have been published to date, all affecting exon six, similar to all *TINF2* mutations described in DKC. The most common germline alteration is c.845G > A, a missense mutation with substitution of guanine to adenine, leading to an amino acid substitution from arginine to histidine at position 282 of the TIN2 protein. Further mutations have been found sporadically that are summarized in Table 1 [17, 19, 34, 41]. Dysfunctional TIN2 results in an impaired protection of telomere ends by the shelterin complex and patients harbor extremely short telomeres. Hence, premature shortening of telomere repeats leads to premature cell senescence [62]. Regarding the variant molecular interactions of the mutant TIN2 protein with its natural ligands, the readers are referred to the respective literature [63–65]. It is still under debate whether the extent of telomere shortening is generally associated with the severity of symptoms [66, 67]. Undoubtedly, extremely short telomeres in RS, even compared to other TBD, correlate with a more severe course as well as the early onset of this disorder.

It is noteworthy that not all alterations in *TINF2* are pathogenic, hence polymorphisms of the gene exist [66]. Conversely, most patients with *TINF2* mutations develop TBDs other than the classical phenotype of RS, including not only 11 to 24% of DKC patients [3, 20, 46], but also HHS, idiopathic pulmonary fibrosis, and aplastic anemia [20, 21, 48, 68, 69]. Even the most frequent *TINF2* mutation, p.R282H, is not specific for RS and it is yet unclear which additional mechanisms lead to its full phenotype. Recently, TIN2 was identified as a modifier of telomerase activity via interaction with TPP1/POT1, representing an alternate mechanism of telomere pathology in TINF2 mutations [70], and failure to restore telomere length during early pregnancy was hypothesized to contribute to very short telomeres in severe variants [67]. In addition, varying gene expression within different tissues may be related to clinical phenotypes [17], but there is still much effort required to elucidate the pathogenic biological mechanisms. Therefore, at the present time, clinical features, a mutation within exon six of *TINF2*, and very short telomeres, e.g. below the first percentile of age, are needed to make an accurate diagnosis.

## Conclusion

We present a comprehensive review on Revesz syndrome by studying previously published patients, in addition to an individual case report first presented here. In summary, RS is characterized by an early onset of exudative retinopathy and bone marrow failure, as well as a very poor survival. Features of the DKC triad, cerebral calcifications, growth retardation, and neurodevelopmental delay are part of the disease in most but not all cases. In addition, we report the first patient from central Europe, who in contrast to all previously published cases, developed hypoplasia of the corpus callosum during early childhood. Since *TINF2* mutations are not restricted to RS, clear definition of clinical features is essential for an adequate diagnosis, but symptoms overlap with other entities. Therefore, clinical registries and further genetic studies are needed to elucidate the molecular background of this rare disorder as well as support the exploration of novel therapeutic strategies aiming at improved telomere maintenance [71].

## Supplementary information

**Additional file 1: Figure 1.** Axial T1-weighted images at the age of one year (a, b) and 3.8 years (c, d) before (a, c) and after (b, d) administration of gadolinium contrast material. Primary high signal intensity was observed within the symmetrical calcified areas in the thalamus and occipital lobe, with foci of pathological enhancement in the front right (arrow) (b), and later reinforced enhancement of the primary high intensity areas (d). There was also, not shown, symmetrical enhancement within the partially calcified nucleus ruber.

**Additional file 2: Figure 2.** Sagittal T2-weighted images demonstrate a reduction in the size of the corpus callosum over time (arrows) due to increasing leukoencephalopathy at one (a) and 3.8 years (b) of age.

**Additional file 3: Figure 3.** Axial T2-weighted MRI images demonstrate initial normal bulbi at age 2.5 months (a), an abnormal right globe with hemorrhage and detachment at age 2.2 years (b), and an abnormal left globe with V-shaped detachment at age 3.8 years (c).

## Data Availability

The datasets used and/or analyzed during the current study are available from the corresponding author on reasonable request.
