# Supplementary figures and images for: Revesz syndrome revisited
Source: Orphanet J Rare Dis. 2020 Oct 23;15:299. doi: 10.1186/s13023-020-01553-y (PMC7583287; doi:10.1186/s13023-020-01553-y)

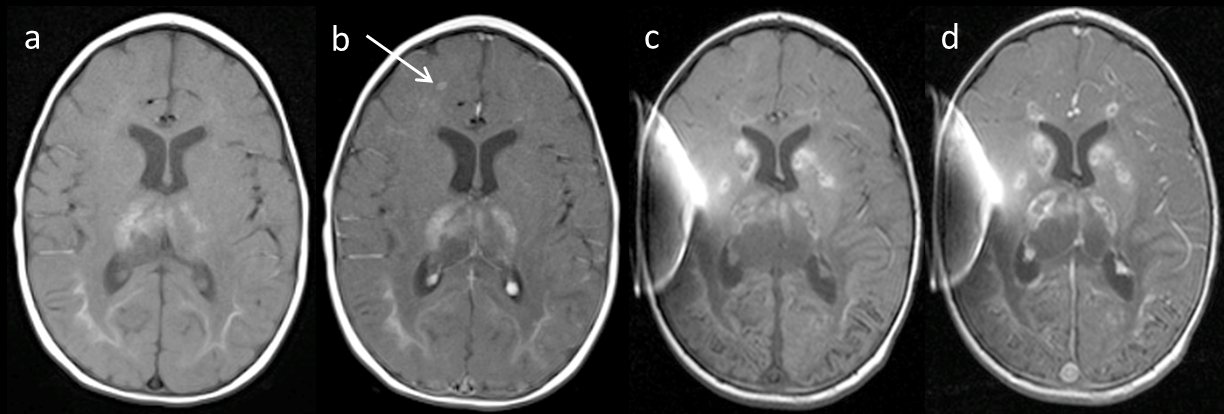

Supplement: Supplementary file 1 — Additional file 1: Figure 1. Axial T1-weighted images at the age of one year (a, b) and 3.8 years (c, d) before (a, c) and after (b, d) administration of gadolinium contrast material. Primary high signal intensity was observed within the symmetrical calcified areas in the thalamus and occipital lobe, with foci of pathological enhancement in the front right (arrow) (b), and later reinforced enhancement of the primary high intensity areas (d). There was also, not shown, symmetrical enhancement within the partially calcified nucleus ruber. [file 13023_2020_1553_MOESM1_ESM.jpg]

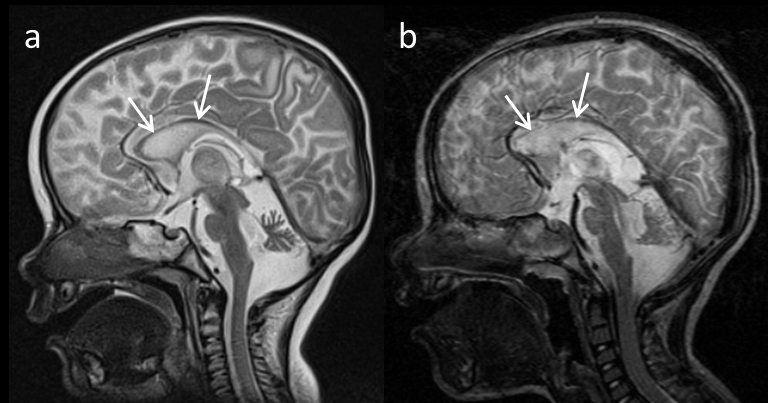

Supplement: Supplementary file 2 — Additional file 2: Figure 2. Sagittal T2-weighted images demonstrate a reduction in the size of the corpus callosum over time (arrows) due to increasing leukoencephalopathy at one (a) and 3.8 years (b) of age. [file 13023_2020_1553_MOESM2_ESM.jpg]

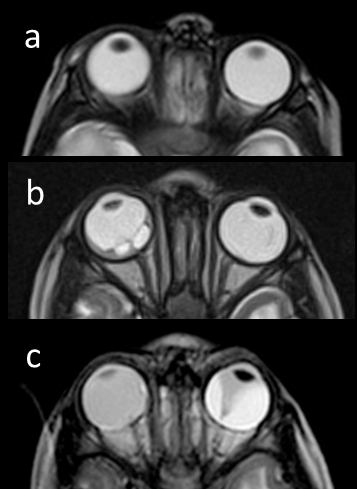

Supplement: Supplementary file 3 — Additional file 3: Figure 3. Axial T2-weighted MRI images demonstrate initial normal bulbi at age 2.5 months (a), an abnormal right globe with hemorrhage and detachment at age 2.2 years (b), and an abnormal left globe with V-shaped detachment at age 3.8 years (c). [file 13023_2020_1553_MOESM3_ESM.jpg]
